# DDX23, an Evolutionary Conserved dsRNA Sensor, Participates in Innate Antiviral Responses by Pairing With TRIF or MAVS

**DOI:** 10.3389/fimmu.2019.02202

**Published:** 2019-09-18

**Authors:** Jie Ruan, Yange Cao, Tao Ling, Peiyi Li, Shengpeng Wu, Dezhi Peng, Yao Wang, Xin Jia, Shangwu Chen, Anlong Xu, Shaochun Yuan

**Affiliations:** ^1^State Key Laboratory of Biocontrol, Guangdong Province Key Laboratory of Pharmaceutical Functional Genes, Department of Biochemistry, College of Life Sciences, Sun Yat-sen University, Guangzhou, China; ^2^School of Life Sciences, Beijing University of Chinese Medicine, Beijing, China; ^3^Laboratory for Marine Biology and Biotechnology, Qingdao National Laboratory for Marine Science and Technology, Qingdao, China

**Keywords:** amphioxus (lancelet), innate antiviral immunity, dsRNA sensors, RNA Helicases, DDX23

## Abstract

DExD/H-box helicases play essential roles in RNA metabolism, and emerging data suggests that they have additional functions in antiviral immunity across species. However, little is known about this evolutionarily conserved family in antiviral responses in lower species. Here, through isolation of poly(I:C)-binding proteins in amphioxus, an extant basal chordate, we found that DExD/H-box helicases DHX9, DHX15, and DDX23 are responsible for cytoplasmic dsRNA detection in amphioxus. Since the antiviral roles of DDX23 have not been characterized in mammals, we performed further poly(I:C) pull-down assays and found that human DDX23 binds to LMW poly(I:C) through its N-terminal region, suggesting that DDX23 is an evolutionarily conserved dsRNA sensor. Knockdown of human DDX23 enhanced the replication of VSV and reduced the activation of the NF-κB and IRF3. Moreover, when stimulated with poly(I:C) or VSV, human DDX23 translocated from the nucleus to the cytoplasm and formed complexes with TRIF or MAVS to initiate downstream signaling. Collectively, this comparative immunological study not only defined DDX23 as an emerging nuclear pattern recognition receptor (PRR) for the innate sensing of an RNA virus, but also extended the essential role of the DExD/H helicase family in viral RNA sensing from mammals to basal chordates.

## Introduction

RNA helicases control nearly every facet of RNA metabolism, including transcription, splicing, miRNA biogenesis, translation, and decay (1–3). Comprising the largest family of helicases, DExD/H-box proteins are found in distinct evolutionary stages and share 12 conserved motifs, including the DExD/H motif (1–3). Although DExD/H-box proteins are most recognized for their roles in RNA metabolism, some have important functions in antiviral defense, including mammalian RIG-I/DDX58, MDA5/IFIH1, DHX9, DHX15, DHX36, DDX17, and DDX41 (4–6). It is well-established that RNA helicases preferentially use different adaptor molecules to trigger antiviral responses. For example, a complex of DDX1, DDX21, and DHX36 pairs with TRIF (7); RIG-I, MDA5, DHX9, and DHX15 use MAVS (6, 8–10); and DHX33 interacts with NLRP3 to form the inflammasome complex following stimulation with RNA (11). Besides interacting with distinct adaptors, RNA helicases are varied in recognition of double-stranded RNA (dsRNA) in different length and structure. For example, RIG-I is a high-affinity receptor for short, blunt-ended dsRNA and 5′ triphosphate (5′ppp) RNA (12, 13), while MDA5 appears to recognize long blunt-ended dsRNA (14). Moreover, MDA5 contributes little to the mDC's response to poly(I:C), while the DDX1-DDX21-DHX36 complex recognizes poly(I:C) specifically in mDCs (7, 15).

In addition to sensing dsRNA, RNA helicases such as DDX41, DHX9, and DHX36 can recognize cytosolic DNA (16–18). DDX41 recognizes intracellular DNA and the bacterial secondary messenger cyclic di-GMP (c-di-GMP) or cyclic di-AMP (c-di-AMP) and forms a complex with STING to signal to TBK1-IRF3 and to activate the interferon response (17, 18). DHX9 selectively binds to CpG-B DNA and is important for NF-κB activation in response to CpG-B, while DHX36 selectively binds to CpG-A DNA and influences IFN-α production and IRF7 nuclear translocation in response to CpG-A (16). In addition to acting as direct RNA/DNA sensors, some helicases, such as DDX60 and DHX36, can promote RIG-I-dependent IFN production in response to dsRNA (19, 20), and some, including DDX24, exert a negative-regulatory effect on RIG-I signaling by binding to adaptor proteins FADD and RIP1, resulting in reduced IRF7 activity. DDX46 inhibits anti-viral responses by recruiting the m6A demethylases ALKBH5 to erase the m6A modification of *MAVS, TRAF3*, and *TRAF6* transcripts, leading to their retention in the nucleus and impaired translation (21, 22). Thus, the DExD/H-box helicase superfamily is a component of the innate immune system important for detecting viral infection.

Although RIG-I-like receptors (RLRs) and the IFN response system are not present in insects, many aspects of innate immunity, as well as many DExD/H-box helicases, are conserved in flies. Insects possess a helicase family that plays a part in combating viral infection (1). The first characterized helicase was DExD/H-box helicase Dicer-2 (Dcr-2), an evolutionarily conserved protein required for RNAi known to perform antiviral functions in *Drosophila* by recognizing double-stranded or structured viral RNAs and cleaving them into 21 nt small-interfering RNAs (siRNAs) to clear viral RNA (23, 24). Two genome-wide RNAi screenings further identified DDX17 (alternatively called Rm62) and DDX6 (alternatively called me31B) as antiviral molecules combating Rift valley fever virus (RVFV), a mosquito-transmitted bunya virus that causes severe morbidity and mortality in humans and livestock (25, 26). Thus, the roles of DExD/H-box helicases in innate immunity are more diverse than previously recognized, and some antiviral helicases likely remain unidentified in both vertebrates and invertebrates.

Amphioxus, as the key transitional species from invertebrates to vertebrates, has an extraordinarily complex innate immune system and possesses a large helicase family (27, 28). To find some evolutionarily conserved DExD/H helicase members participating in antiviral responses across species, a series of poly(I:C) binding assays were performed to identify the poly(I:C) binding proteins in amphioxus, and three evolutionarily conserved DExD/H-box helicases, DHX9, DHX15, and DDX23, were identified. Since antiviral functions of DHX9 and DHX15 have recently been reported in mammals (6, 9, 10) and the predicted subcellular localization of DDX23 is the nucleus, we conducted cross-species analyses of DDX23, and found that it is an evolutionarily conserved dsRNA sensor involved in antiviral immunity.

## Materials and Methods

### Cell Culture and Biological Reagents

Human embryonic kidney cells (HEK293T) and A549 cells were maintained in Dulbecco's modified Eagle's medium (DMEM; Invitrogen) supplemented with 10% FBS, 10 mM Hepes and 2 mM L-glutamine. Poly(I:C) and Poly(dA:dT) were purchased from InvivoGen. The silver staining kit and Lipofectamine 2000 were purchased from Invitrogen. The following antibodies were used for immunoprecipitation and/or immunoblotting: anti-DDX23 (abcam), anti-TRIF (Cell signaling technology, CST), MAVS (abcam), TBK1, p-TBK1, p38, p-p38, p65, p-p65, IRF3, p-IRF3 (CST). Anti-HA, anti-FLAG beads, poly(I:C) agarose, poly(C) agarose, and anti-VSV-G were purchased from Sigma.

### Culture of Amphioxus Intestinal Cells

Adult Chinese amphioxus *B. belcheri* were collected from Zhanjiang, China and reared in aerated sea water with algae. Three days before dissection, amphioxus were transferred to sea water without algae in order to evacuate the intestine. On the day prior to dissection, sea water was filtered with a 0.45 μm filter to remove large particles, and then 10 mg/ml penicillin was added for intestine sterilization.

After amphioxus were anesthetized, the amphioxus intestines were extracted, dissected into pieces, and digested for 2 h at 23°C with 1% collagenase type II (Gibco). The cells were suspended in a culture medium [DMEM high glucose (Gibco), DMEM/F12 (Gibco), and Leiboviz's L15 (Gibco) at a ratio of 1:1:1] containing 10% fetal bovine serum (Gibco) and penicillin-streptomycin (Gibco). The cell suspension was cultured in a 35 mm tissue culture dish at a density of five intestines per dish at 23°C.

### Generation of Poly(I:C) Agarose and Isolation of Poly(I:C) Binding Proteins From Amphioxus Intestine Cells

To generate poly(I:C) agaroses, poly(C) agarose (Sigma) were mixed in 5 mg/ml poly(I) (Sigma) in buffer of 50 mM Tris (PH 7.0) and 150 mM NaCl. The mixture was mixed gently 12 h at 4°C. Next, the beads were washed twice with TBS and twice with TAP lysis buffer (50 mM Tris pH 7.5, 150 mM NaCl, 1 mM EDTA, and 0.2% NP-40, protease inhibitors).

Amphioxus primary cells were lysed in TAP lysis buffer and protein concentrations were measured with a Bradford assay. Cell extracts were added to the poly(I:C) agarose or control poly(C) agarose followed by 12 h at 4°C. Beads were washed extensively with lysis buffer, re-suspended in SDS-PAGE sample buffer, separated on an SDS-PAGE and stained with silver. Entire gel lanes from the poly(I:C) or the controls were excised from the gels and analyzed by nano LC-MS/MS. Two separated experiments were performed.

### Cloning of bbeDHX15 and bbeDDX23 cDNAs

The draft genome of *B. belcheri* and the related analysis tools can be accessed at http://mosas.sysu.edu.cn/genome/. Partial sequences of bbeDHX15, bbeDDX23 were obtained by RT-PCR using primer pairs derived from bbeDHX15 (ID: 135710F) and bbeDDX23 (ID: 054280F). Then 5′RACE and 3′RACE using gene specific primers derived from bbeDHX15 and bbeDDX23 were performed according to the GeneRACE™ Kit (Invitrogen). ID numbers are the protein ID for sequences identified in the website http://genome.bucm.edu.cn/lancelet/.

### Poly(I:C) Transfection of Amphioxus Intestine Cells and qRT-PCR Assays

To test the expression pattern of amphioxus DExD/H helicases upon poly(I:C) stimulation, the primary amphioxus intestine cells were transfected with poly(I:C) (Sigma-Aldrich) at a final concentration of 3 μg/ml and collected at 0, 2, 4, 6, 12, and 24 h post-poly(I:C) transfection. Then qRT-PCR assays were conducted using primer pairs:

BbeDHX9-F: 5′GACTACCAGGAATACTTTGAG3′

BbeDHX9-R: 5′CACCACATGATGCTCCACCTT3′

BbeDHX15-F: 5′GGTCGTGGTGTCTACTAACAT3′

BbeDHX15-R: 5′GGTACGTCTGGTCCTGCATCT3′

BbeDDX23-F: 5′CCCAGCAGATCGAGGAGGAGA3′

BbeDDX23-R: 5′CACATCTGGCTCGAAACCCAT3′

### RNA Sequencing

The RNA sequencing studies were performed with A549 cells transfected with scrambled siRNA (siControl) or siDDX23 targeting the *DDX23* gene for 3 days following 12 h infection with VSV (MOI = 3) or being uninfected. Total RNA was isolated using TRIZOL Reagent (Invitrogen). A cDNA library was constructed using the NEBNext UltraTM RNA Library Prep Kit for Illumina (NEB, USA) following the manufacturer's recommendations and then sequenced with the HiSeq 2000 system (Illumina Inc.). The expression of transcripts was quantified as reads per kilobase of exon model per million mapped reads (RPKM). Subsequently, gene enrichment analysis was conducted using the DAVID tool. The raw data of RNA sequencing has been deposited to GEO public database (accession number GSE132847).

### Poly(I:C) Transfection of Human A549 Cells and qRT-PCR Assays

To test the expression pattern of several cytokines upon poly(I:C) stimulation when human DDX23 was knockdown, A549 cells were transfected with poly(I:C) at a final concentration of 3 μg/ml and collected at 0, 2, 4, 6, and 12 h post-poly(I:C) transfection. qRT-PCR assays were then performed using the primer pairs suggested by http://pga.mgh.harvard.edu/primerbank/.

hIFNB1-F: 5′GCTTGGATTCCTACAAAGAAGCA3′;

hIFNB1-R: 5′ATAGATGGTCAATGCGGCGTC3′

hCXCL10-F: 5′GTGGCATTCAAGGAGTACCTC3′

hCXCL10-R: 5′TGATGGCCTTCGATTCTGGATT3′

hIL6-F: 5′ACTCACCTCTTCAGAACGAATTG3′

hIL6-R: 5′CCATCTTTGGAAGGTTCAGGTTG3′

hTNFα-F: 5′CCTCTCTCTAATCAGCCCTCTG3′

hTNFα-R: 5′GAGGACCTGGGAGTAGATGAG3′

hIFNA2-F: 5′GCTTGGGATGAGACCCTCCTA3′

hIFNA2-R: 5′CCCACCCCCTGTATCACAC3′

### Measurement of Cytokine Production

Human A549 cells were transfected with indicated siRNAs for 48 h. Cells were then transfected with 3 μg/ml poly(I:C). The concentrations of IFN-β, IL-6, and RANTES in the culture supernatants were measured by ELISA according to the manufacturer's protocol. IFN-β ELISA kits were purchased from PBL Interferon-Source. IL-6 and RANTES ELISA kits were purchased from R&D Systems, Inc.

### Purification of myc-tagged bbeDHX15, bbeDDX23, and HA-tagged hsDDX23

For HA or myc elution, HEK293T cells were transfected with the indicated HA or may-tagged constructs. After 24–48 h, cells were collected in PBS and dounce homogenized in a hypotonic lysis buffer containing 10 mM NaCl, 1.5 mM MgCl_2_, 10 mM Tris pH 7.5, and Complete protease inhibitor cocktail (Roche). Lysates were cleared by centrifugation and immunoprecipitated with anti-HA or anti-c-myc affinity gel (Sigma) 4 h overnight. Immunoprecipitates were washed once with wash buffer 1 (20 mM HEPES pH 7.9, 420 mM NaCl, 1.5 mM MgCl_2_, 0.2 mM EDTA, 25% glycerol, Complete protease inhibitor cocktail) and four times with wash buffer 2 (20 mM Tris pH 7.4, 20% glycerol, 0.2 mM EDTA, 300 mM NaCl, 0.1% NP-40, Complete protease inhibitor cocktail) and rotated at least 2 h in wash buffer 2. Samples were eluted with 5 μg/ul 3 × HA or c-myc peptide (Sigma) according to the manufacturer's instructions.

### *In vitro* Pull-Down and Immunoblotting Assay

Lysates from HEK293T cells transfected with the indicated expression plasmids were incubated with anti-HA or anti-myc beads. Proteins were eluted from beads and incubated for 4 h with poly(I:C) agarose in the presence or absence of poly(I:C) or other competitors. Centrifugation was followed by incubation with beads, and beads were analyzed by immunoblotting with anti-HA or anti-myc.

### Co-immunoprecipitation

HEK293T cells in six-well dishes were transfected with a total of 4 μg DNA plasmids. At 24–36 h post-transfection, the whole cell extracts were prepared in IP lysis buffer [50 mM Tris, pH 7.4, 150 Mm NaCl, 1% NP-40, 0.5% deoxycholic acid sodium salt and cocktail protease inhibitor (Roche)] and incubated with primary antibodies at 4°C for 4 h, then incubated with Protein G Sepharose (Roche) at 4°C overnight. On the second day, the beads were washed three times with lysis buffer, mixed with 4 × SDS loading buffer and boiled for 2 min. The analysis was conducted using SDS-PAGE followed by a Western blot, using the ECL protocol (Amersham Pharmacia).

### Luciferase Reporter Assay

HEK293T cells seeded on 48-well plates were transfected with 100 ng of the luciferase reporter vector controlled by the IFN-β promoter, or the NF-κB binding motif, or the ISRE motif together with indicated expression vectors or empty control vectors. A total of 2 ng of Renilla-luciferase reporter gene was transfected simultaneously for the internal control. Twenty-four hours later, the luciferase activity in the total cell lysate was detected with the Dual Luciferase Reporter Assay System (Promega).

### Subcellular Fractionation

Cell fractionation assays were performed according to protocols of the NE-PER Nuclear and Cytoplasmic Extraction Kit (Thermo Scientific). The purity of nuclear and cytoplasmic extracts was assessed by immunoblotting with anti-lamin B (proteintech) and either anti-α-tubulin or anti-GAPDH antibodies (proteintech), respectively.

### Phosphorylation Assay

A549 cells were lysed in RIPA buffer supplemented with protease and phosphatase inhibitors after a 0, 4, 8, and 12 h stimulation with low molecular weight poly(I:C) at a final concentration of 3μg/ml. Lysates were resolved by 10% SDS-PAGE and blotted with Abs recognizing indicated phosphorylated proteins.

## Results

### Isolation of Poly(I:C)-Binding Proteins in Amphioxus Intestinal Cells Identified DHX9, DHX15, and DDX23

**Figure 1:**
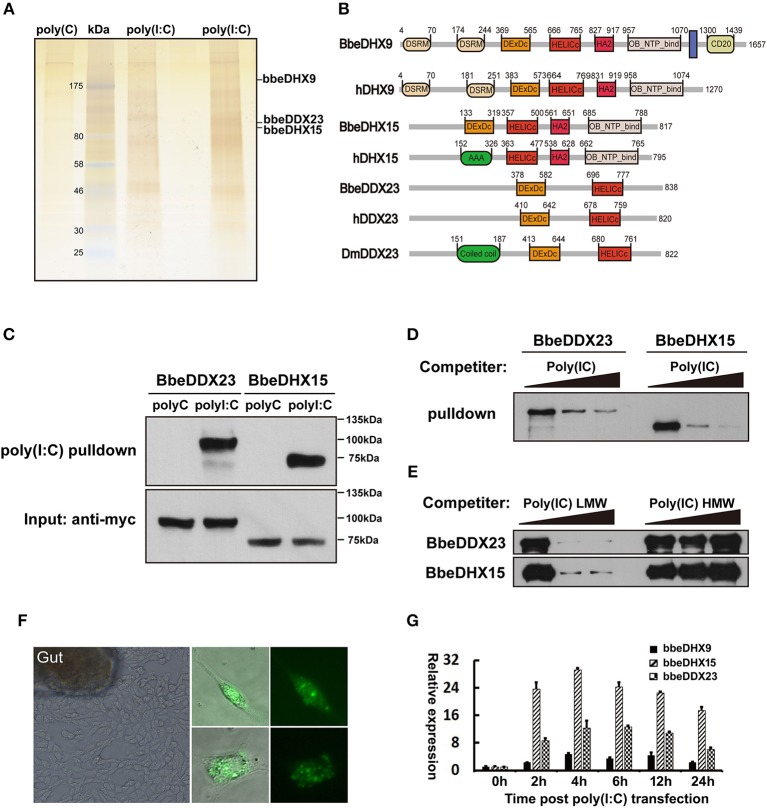
Isolation of poly(I:C) binding proteins from amphioxus intestinal cells. **(A)** Silver stained poly(I:C)-associated proteins from amphioxus intestinal cells purified with the poly(I:C) agarose or control poly(C) agarose. Proteins identified by LC-mass spectrometry. Two poly(I:C) lines indicated samples from two separated experiments. **(B)** The comparison of protein architectures between *Drosophila*, amphioxus and human DDX23, DHX9, and DHX15. All these helicases have highly conserved DEXDc (DEAD-like helicases superfamily) and HELICc (helicase superfamily c-terminal) domains. **(C)** Pull-down assays were performed by incubating purified myc-bbeDHX15 or myc-bbeDDX23 with the poly(I:C) or poly(C) agarose. Bound proteins were analyzed by immunoblotting with anti-myc. **(D)** The mixture of myc-bbeDDX23 or myc-bbeDHX15 and poly(I:C) agaroses were incubated with or without free poly(I:C) at 0, 150, or 500 μg/ml. Bound proteins were analyzed by immunoblotting with anti-myc. **(E)** Pull-down competition assays using purified myc-bbeDDX23 or myc-bbeDHX15 in the presence of increasing amounts (0, 1,000, 3,000 μg/ml) of uncoupled low molecular weight (LMW) poly(I:C) or high molecular weight (HMW) poly(I:C). **(F)** Morphology of primary amphioxus intestinal cells in both resting and upon FITC-labeled poly(I:C) transfection. **(G)** Expression profiles of bbeDHX9, bbeDHX15, and bbeDDX23 were determined by RT-PCR using poly(I:C) transfected amphioxus intestinal cells. Data are shown as the means ± standard deviations of three samples per treatment. Values were considered to be significant when *p* < 0.05. The results were confirmed by at least three separate experiments.

To systematically identify previously unknown dsRNA sensors in amphioxus, we designed a proteomic screen using poly (I:C) agaroses and protein pull-down experiments. First, we cultured primary amphioxus intestine cells, as the digestive system is thought to comprise the major immune organs of amphioxus and contain many immune-related cells, including lymphocyte-like, monocyte-like, and macrophage-like cells (29, 30). Whole amphioxus intestine cell extracts were added to the poly(I:C) agaroses or control poly(C) agaroses followed by incubation at 4°C overnight. The proteins bound to poly(I:C) were separated by gel electrophoresis and stained with silver (Figure 1A). Entire gel lanes from the poly(I:C) or the controls were excised from the gels and analyzed by LC-mass spectrometry (LC-MS). We found the known dsRNA sensors, DHX9 and DHX15, which validated our method of isolating dsRNA-binding proteins from amphioxus intestinal primary cells (Figure 1B). In addition to DHX9 and DHX15, the poly(I:C) pulled down a new member of the DExD/H-box helicase family, DDX23, a spliceosome component required for splicing of nuclear pre-mRNA (Figure 1B).

### The Immune Functions of Amphioxus DHX9, DHX15, and DDX23

Amphioxus has a large DExD/H helicase family including DHX9, DHX15, and DDX23, which exhibited high sequence identity and domain conservation to their human counterparts (Supplemental Figure 1). To validate the binding of amphioxus DHX9, DHX15, and DDX23 to poly(I:C), we cloned bbeDHX15 and bbeDDX23 from the Chinese amphioxus *Branchiostoma belcheri* (bbe) cDNA library, and prepared recombinant myc-tagged bbeDHX15 and bbeDDX23 by transfecting HEK293T cells with plasmids encoding the recombinant proteins and purifying them with anti-myc beads. Each purified helicase was incubated with poly(I:C) or poly(C) agaroses and was found to bind poly(I:C) but not polyC control, confirming our previous MS analyses (Figure 1C). Evidence for the specific binding of bbeDHX15 and bbeDDX23 to poly(I:C) was strengthened by efficient competition with increasing amounts of uncoupled poly(I:C) (Figure 1D). Interestingly, only low molecular weight (LMW) poly(I:C), a well-known RIG-I agonist, could block the binding of poly(I:C) agarose to bbeDHX15 and bbeDDX23, suggesting that bbeDHX15 and bbeDDX23 may preferentially bind to short dsRNA molecules (Figure 1E). We also carried out RT-PCR analysis using poly(I:C) transfected amphioxus intestine cells and found that the expression of bbeDHX9, bbeDHX15, and bbeDDX23 increased more than 10-fold in response to poly(I:C) stimulation (Figures 1F,G), further indicating that these helicases may play essential roles in cytosolic dsRNA induced immune responses in amphioxus.

### DDX23 Is an Evolutionarily Conserved dsRNA Sensor

**Figure 2:**
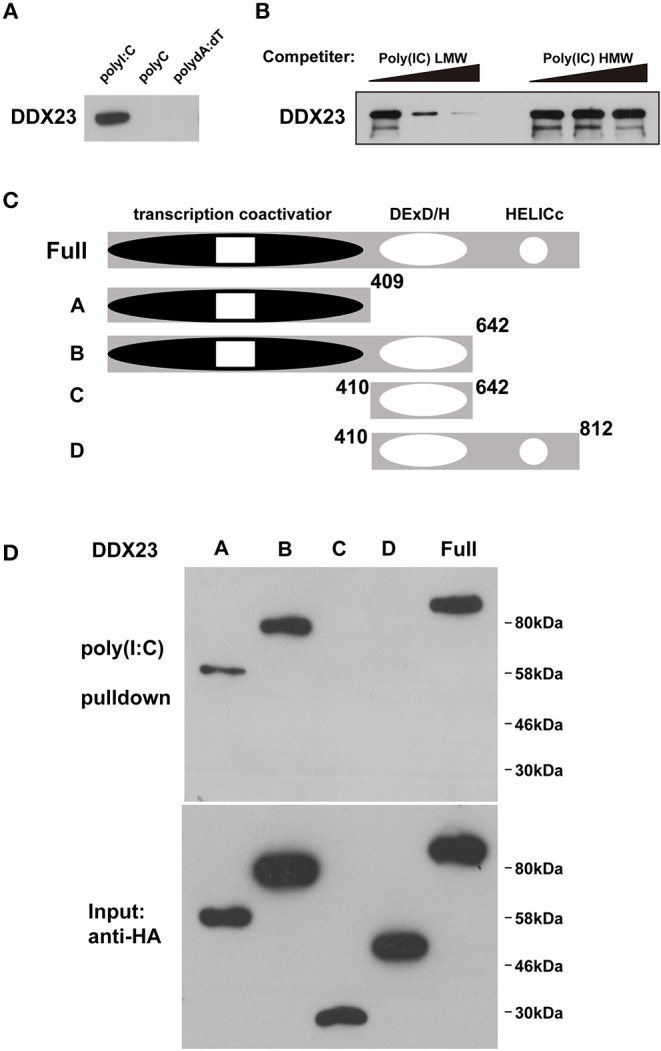
DDX23 is an evolutionarily conserved dsRNA sensor across species. **(A)** Pull-down assays were performed by incubating purified human HA-DDX23 with poly(I:C) agaroses. Bound proteins were analyzed by immunoblotting with anti-HA. Poly(C) and poly(dA:dT) were used as control. **(B)** The mixture of human HA-DDX23 and poly(I:C) agaroses were incubated with or without free LMW or HMW poly(I:C) at 0, 150, or 500 μg/ml. Bound proteins were analyzed by immunoblotting with anti-HA. **(C)** Schematic representations of DDX23 and its serial deletion mutants. Full: DDX23 full size; A: N terminal transcription coactivator domain of DDX23; B: N terminal and the DExD/H region of DDX23; C: DExD/H region of DDX23; D: DExD/H and HELICc domains of DDX23. Numbers denote amino acid residues. **(D)** Truncated HA fusions were purified (lower panel) and individually incubated with poly(I:C) agaroses. Bound proteins were analyzed by immunoblotting with anti-HA (upper panel).

Gene knockdown technology has not been well-established, and the antiviral effectors have not been identified in amphioxus, raising a technological challenge to further study these dsRNA sensors in amphioxus at present. As the antiviral activity of mammalian DHX9 and DHX15 has recently been revealed (6, 9, 10), we conducted functional analyses of mammalian DDX23 and *Drosophila* DDX23. We first performed poly(I:C) binding assays to investigate whether DDX23 is an evolutionarily conserved dsRNA sensor. Results showed that, similar to bbeDDX23, human DDX23 and *Drosophila melanogaster* DDX23 (DmDDX23) could also bind to poly(I:C), but not dsDNA and poly(C) (Figure 2A and Supplemental Figure 2A). By performing competition experiments, we found that increasing amounts of LMW poly(I:C) efficiently blocked the binding of poly(I:C) agarose to human DDX23 (Figure 2B), further suggesting that RNA binding capabilities and specifications of DDX23 are evolutionarily conserved. To map the poly(I:C)-binding site of human DDX23, we prepared truncated versions of DDX23 and performed poly(I:C) pull-down experiments (Figure 2C). Then the N-terminal region, but not the DExD/H-box nor HELICc domains of DDX23, was shown to bind poly(I:C) (Figure 2D).

### DDX23 Is Involved in Antiviral Responses

**Figure 3:**
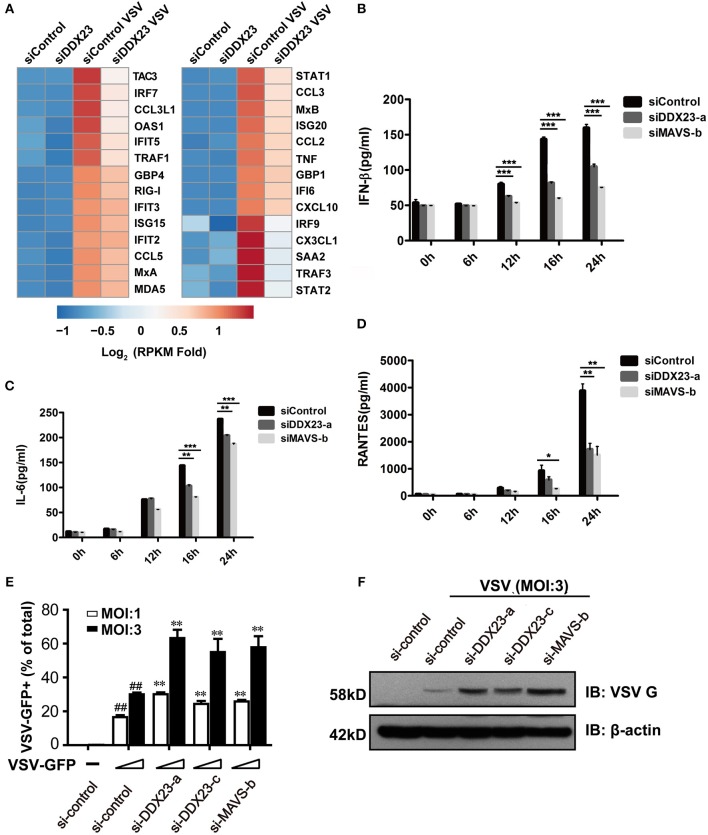
Human DDX23 plays an important role in innate immune responses to poly(I:C) stimulation and VSV infection. **(A)** Heatmap of RNA-seq analysis. A549 cells were transfected with siRNA (control) or with siRNA targeting DDX23 (si-DDX23) for 3 days and then uninfected or infected with VSV (MOI = 3) for 12 h. Heatmap was made by calculating log_2_ [(infected RPKM)/(control RPKM)]. **(B–D)** ELISA of IFN-β, IL-6, and RANTES production by indicated cells stimulated with LMW poly(I:C) delivered with Lipofectamine 2,000 for 24 h. Mean ± SEM; *p < 0.05, **p < 0.01, ***p < 0.001, Student's *t*-test. **(E)** Relative percentage of VSV-eGFP-infected cells (MOI = 1 or 3) transfected with indicated siRNAs 24 hpi. Mean ± SEM; *p < 0.05, **p < 0.01, Student's *t*-test. ^*##*^*p* < 0.01 on VSV-infected si-control column compared to uninfected si-control. **(F)** A549 cells were transfected with the indicated siRNAs and infected with VSV-eGFP for 16 h. Viral protein was monitored by immunoblotting (MOI = 3).

To comprehensively reveal how DDX23 participates in the response to RNA virus infection, we knocked down human DDX23 with siRNA in A549 cells and performed an RNA-seq analysis to compare the transcriptional changes induced by VSV infection in scrambled siRNA (siControl) and DDX23 knockdown (siDDX23) A549 cells (Supplemental Figure 2B and Figure 3A). Results showed that a cluster of IFN-stimulated genes (ISGs) and inflammation-related genes were markedly deduced in DDX23 silencing cells after VSV infection (Figure 3A). Moreover, differentially expressed genes of siControl vs. siDDX23 cells by VSV infection showed enrichment for innate antiviral immune responses such as JAK-STAT, NF-κB, and Toll-like receptor signaling pathways, suggesting that DDX23 plays an important role in RNA virus-triggered type I IFN signaling (Supplemental Figure 3). In addition, the effect of DDX23 was extended to poly(I:C). Knockdown of DDX23 resulted in ~40% reduction in IFN-β production when responding to poly(I:C) (Figure 3B). Knockdown of DDX23 also resulted in reduced production of IL6 and RANTES in response to poly(I:C) (Figures 3C,D). Quantitative RT-PCR (qRT-PCR) assays confirmed that transcription of *IFN-*β*, IFN-*α*, TNF-*α*, IL6, RANTES*, and *CXCL10* in response to poly(I:C) or VSV infection-induced upregulation of *RIG-I, Mda5, MxA, OAS1, and IFIT2* is significantly decreased when the expression of DDX23 was knockdown (Supplemental Figures 2C–M). In contrast, HSV-1-induced transcription of *IFN-*β and *IFIT2* was comparable between DDX23 knockdown and control cells (Supplemental Figures 2N,O). To further examine the involvement of DDX23 in eliminating live viruses, human A549 cells were transfected with siRNA for 48 h, and then infected with VSV-eGFP. Immunoblotting for VSV G glycoprotein and FACS analysis of GFP-positive cells both showed that knockdown of DDX23 significantly enhanced the replication of VSV in A549 cells (Figures 3E,F). In contrast, over-expressed DDX23 significantly reduced the replication of VSV in A549 cells (Supplemental Figure 4A).

### Intracellular Localization of Human DDX23

**Figure 4:**
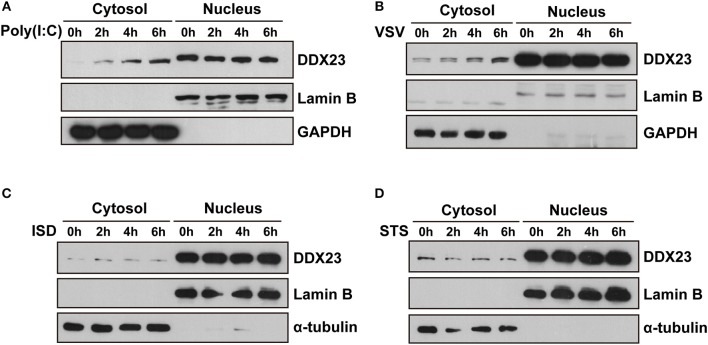
DDX23 is translocated to the cytoplasm after poly I:C or VSV stimulation. **(A–D)** Immunoblot analysis of endogenous DDX23 in the cytoplasmic and nuclear fractions of A549 cells treated with poly(I:C), VSV, ISD, or staurosporin (STS) for the indicated times. Data are representative of at least two independent experiments.

Since most helicases play essential roles in RNA metabolism and are located in the nucleus, we studied the intracellular localization of the human DDX23 protein using subcellular fractionation analysis. We found increased cytoplasmic distribution of DDX23 at 2 h post-stimulation with poly(I:C) or VSV and sustained for 6 h (Figures 4A,B). However, neither interferon stimulatory DNA (ISD) nor staurosporin (STS) triggered such obvious translocation of DDX23 (Figures 4C,D), which strongly suggested that the redistribution of DDX23 is specifically to dsRNA or RNA virus treatment.

### Human DDX23 Binds With TRIF or MAVS to Transfer Antiviral Signals

**Figure 5:**
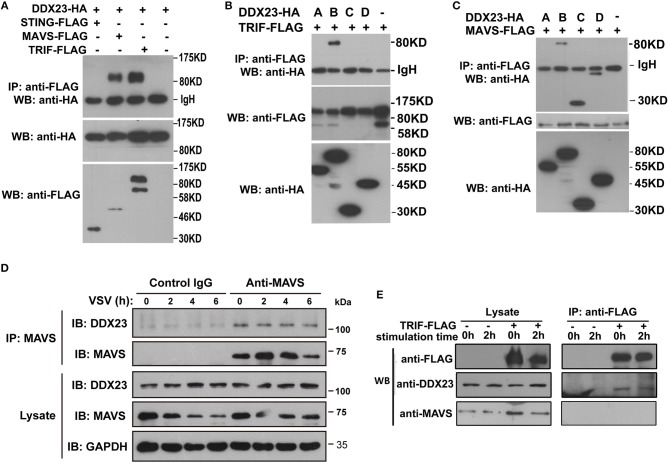
Interactions among DDX23, TRIF, and MAVS. **(A)** HA-DDX23 was co-expressed with FLAG-TRIF, FLAG-MAVS, FLAG-STING, or control vectors, and immunoprecipated with anti-FLAG beads. Bound proteins were analyzed by immunoblotting with anti-HA. **(B)** HA-tagged DDX23 full-size or truncated expression plasmids were co-expressed with FLAG-tagged TRIF in 293T cells. After incubation with anti-FLAG beads, the bound proteins were analyzed by immunoblotting with anti-HA. **(C)** HA-tagged DDX23 truncations were individually co-expressed with FLAG-MAVS in 293T cells. After incubation with anti-FLAG beads, the bound proteins were analyzed by immunoblotting with anti-HA. **(D)** Whole-cell lysates from A549 cells, with or without VSV infection, were incubated with anti-MAVS and Protein G beads. Bound proteins were analyzed by immunoblotting with anti-DDX23 or anti-MAVS antibodies. **(E)** A549 cells were transfected with FLAG-tagged TRIF for 24 h. Whole-cell lysates from FLAG-tagged TRIF expressed A549 cells, with or without poly(I:C) stimulation, were incubated with anti-FLAG and Protein G beads. Bound proteins were analyzed by immunoblotting with DDX23 and MAVS antibodies. All data are representative of at least two independent experiments.

To reveal how human DDX23 initiates antiviral signals, we performed a series of Co-IP assays to determine which adaptor molecule may be used by DDX23 for signal transduction. Results showed that DDX23 interacts with both TRIF and MAVS, but not with STING (Figure 5A). To map the adaptor binding sites of DDX23, we conducted Co-IP assays with truncated versions of DDX23 and TRIF and observed that the N terminal and DExD/H region are both critical for the interaction between human DDX23 and TRIF (Figure 5B). Likewise, truncated versions of DDX23 were co-expressed with MAVS and found that DDX23 binds MAVS via its DExD/H box (Figure 5C). We further investigated the potential interaction between endogenous human DDX23 and MAVS in A549 cells. We performed anti-MAVS immunoprecipitation (IP) experiments in A549 cells and found that endogenous DDX23 interact with MAVS in both resting and VSV-infected A549 cells (Figure 5D). Since the TRIF antibody purchased from CST is hard to immunoprecipate the endogenous TRIF, we had to overexpress FLAG-tagged TRIF in A549 cells and performed an anti-FLAG IP experiment. FLAG-tagged TRIF was shown to form a complex with endogenous DDX23 both in resting and poly(I:C) stimulated cells (Figure 5E). However, the endogenous MAVS could not be identified in the TRIF-DDX23 complex, suggesting that DDX23 could not associate with MAVS when interacting with TRIF. Thus, DDX23 could transfer its anti-viral signals via an TRIF-dependent or MAVS-dependent manner.

### DDX23 Activates NF-κB and IFN-Response Signal Transduction Pathways Upon Poly(I:C) Stimulation

**Figure 6:**
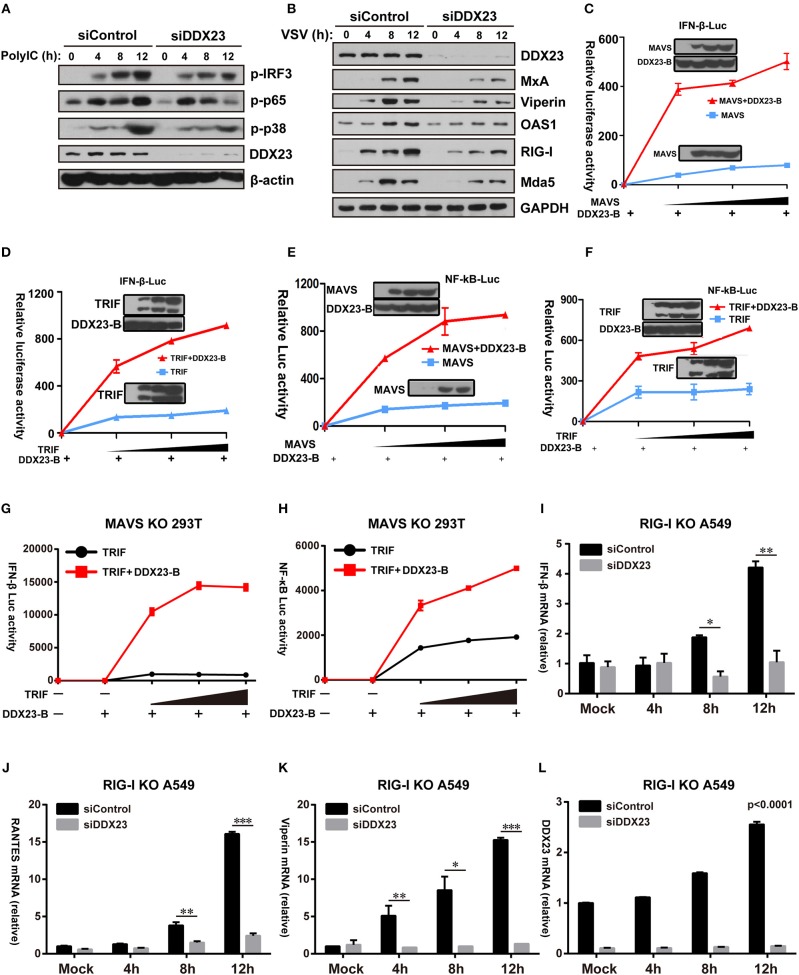
Human DDX23 responses to poly(I:C) by activating the NF-κB and IFN responses. **(A,B)** Western blot analysis of indicated proteins in A549 cells transfected with control siRNA or siDDX23 and stimulated for 4, 8, and 12 h with low molecular weight poly(I:C) or VSV. Data are representative of at least two independent experiments. **(C–F)** Luciferase activity in A549 cells transfected with IFN-β (up) or NF-κB (down) promoter-driven luciferase reporters and together with plasmid DDX23 and increasing amount of plasmid expressing MAVS or TRIF. **(G,H)** MAVS KO 293T was transfected with IFN-β or NF-κB promoter luciferase reporter (100 ng) and together with plasmid DDX23-B plus increasing amount of plasmid expressing MAVS or TRIF (50, 100, or 200 ng). **(I–L)** qRT-PCR analyses of *IFN-*β*, RANTES, Viperin*, and *DDX23* expression levels in RIG-I KO A549 cells transfected with indicated siRNAs and 48 h later with 3 μg/ml of low molecular weight polyI:C. The data are normalized with human β-actin. Similar results were obtained from three independent experiments. One representative experiment is shown (Mean ± SD, *p < 0.05, **p < 0.01, ***p < 0.001, Student's *t*-test).

It was previously shown that the overexpression of MAVS or TRIF in HEK293T cells resulted in robust activation of IRF3 and NF-κB (8, 31). We investigated whether downstream signaling of DDX23 induced by poly(I:C) involved IRF3 and NF-κB activation. Scrambled siRNA (siControl) and DDX23 knockdown (siDDX23) A549 cells were stimulated with LMW polyI:C or VSV, and total cell extracts were prepared and analyzed by SDS-PAGE followed by Western blotting (Figures 6A,B). The phosphorylation of IRF3 was decreased in DDX23-knockdown cells at 12 h after polyI:C stimulation. Compared to control siRNA, in response to polyI:C stimulation, the phosphorylation of p65 and p38 were also reduced in DDX23 knockdown cells (Figure 6A). Moreover, DDX23 silencing did not alter the expression of key signaling molecules like TBK1, MAVS and MAVS but decreased the induction of several ISGs such as MxA, Viperin, OAS1, RIG-I, and Mda5 after VSV infection (Supplemental Figure 4B and Figure 6B). These data strongly suggested that DDX23 is involved in activating the NF-κB and IFN-response upon dsRNA or RNA virus stimulation.

Given that DDX23 is a known component of the cellular mRNA splicing machinery, we further explored whether DDX23 influences the alternative splicing of known antiviral factors during viral infection. As depicted in Supplemental Figures 4C,D, knockdown of DDX23 did not alter MxA mRNA splicing but inhibited the transcriptional upregulation of MxA in response to VSV infection, further confirming that DDX23 works in promoting ISG expression independently of cellular mRNA splicing.

Further reporter assays were performed to reveal whether DDX23-TRIF and DDX23-MAVS complexes are both crucial for DDX23 to transfer its anti-viral signals. Results showed that neither full length DDX23 nor the truncated mutants alone could activate the NF-κB and IFN-responses (Supplemental Figure 5A). However, when co-expressed with TRIF or MAVS, the truncated mutant DDX23-A and DDX23-B, but not DDX23-C and DDX23-D, could mount the activation of NF-κB and IFN-responses mediated by TRIF or MAVS (Supplemental Figures 5B–E). In particular, DDX23-B can mount the activation of NF-κB and IFN-responses mediated by MAVS or TRIF in a dose dependent manner (Figures 6C–F). Although DDX23-C could interact with MAVS, it lacks the N-terminal region, suggesting that the N-terminal region of DDX23 is not only required for the dsRNA recognition, but is also crucial for its downstream signaling. Moreover, DDX23-B also significantly elevated TRIF-mediated NF-κB and IFN-responses in MAVS deficient 293T cells which provides strong evidence that DDX23-TRIF transfer anti-viral signals in a MAVS-independent manner (Supplemental Figure 5F and Figures 6G,H).

The above-mentioned data demonstrate that DDX23 may preferentially bind to short dsRNA molecules (Figure 2B), a well-known feature for RIG-I. We then explored whether DDX23-mediated signaling is dependent on RIG-I. siRNA knockdown of DDX23 significantly reduced the residual *Ifnb1, Rantes, Viperin* and *Ddx23* mRNA expression following LMW polyIC stimulation in RIG-I deficient A549 cells, indicating that DDX23-TRIF and DDX23-MAVS are independent of RIG-I to activate downstream signaling (Figures 6I–L).

## Discussion

### DExD/H Helicases Play Essential Antiviral Roles Across Species

The key steps in the innate immune responses are the rapid detection of invading pathogens and the induction of type I interferons (IFNs) and/or pro-inflammatory cytokines (5). Viral RNAs, which are generated during RNA virus replication in host cells, can be recognized by nucleic acid sensors that trigger type I IFNs or inflammatory cytokine responses (32). The mammalian RLR family, including RIG-I, MDA5, and LGP2, is well-characterized with respect to dsRNA sensors, which can recognize different types of RNA molecules and protect against different viral pathogens. However, studies have been unable to recover RLR genes from arthropods, despite the availability of multiple genome sequences (33). Unlike MDA5/LGP2, which are well-conserved in vertebrates, a clearly identifiable RIG-I homolog was lost in some fish species and is also missing in the chicken genome (34). In addition, no evidence for canonical RLR-pathway effector molecules (interferons or pro-inflammatory cytokines) has been reported outside of vertebrates (35). Thus, besides the RIG-I-IFN pathways, metazoan cells should develop alternative mechanisms of defense against viral invasion. Indeed, ever-increasing evidence suggests that other members of DExD/H helicases play critical roles in antiviral innate immune responses. A study has demonstrated that Dicer, another RIG-I-like DExD/H helicase, senses viral nucleic acids in the *Drosophila* innate immune system (23). Many other helicase members, including DDX1, DHX9, and DHX36, have been demonstrated to be involved in antiviral responses in mammals (6, 7, 20). Similarly, three helicase members, DDX6, DDX17, and DDX23 were shown to restrict the replication of RVFV in *Drosophila*, in which DDX17 binds the essential stem loop in bunya viral RNA to combat infection (25, 26). Here, we identified amphioxus DHX9, DHX15, and DDX23 to be dsRNA sensors, further suggesting the roles of DExD/H helicase family members in dsRNA sensing in lower species. The genomic analysis of the DExD/H helicases showed that amphioxus possesses a comparatively complex DExD/H helicase family, including orthologs of vertebrate DDX1, DDX3, DDX17, DDX24, DDX41, and DHX33 (Supplemental Figure 1). Similar to DHX9, DHX15, and DDX23, these identified DExD/H helicases in amphioxus showed high conservation in protein architecture and sequence identities (Supplemental Figure 1). Thus, the roles played by DExD/H helicases in antiviral innate immune responses are conserved across species and were broader than previously thought.

### DDX23 Functions as an Emerging Nuclear Viral RNA Sensor

A previous study has shown that DmDDX23 restricts the replication of RVFV in *Drosophila* (26). Here we further demonstrated that the ability of DDX23 to recognize LMW poly(I:C) is well-conserved from insects to mammals, suggesting that the RNA binding specification of DDX23 is evolutionarily conserved. This may be due to the arm race between host and viruses over a long evolutionary period. As for the negative-strand RNA viruses, which include some of the most dangerous human pathogens such as the Ebola and the Rabies virus, are prone to generate a great amount of short RNA molecules during their replication as the strategy for evading innate immune detection (36, 37). Thus, we can speculate that the host may have evolved redundant innate sensors with short RNA preference to cover a broad spectrum of RNA viruses, such as DDX23 and the well-known RIG-I which also has high-affinity for short dsRNAs.

In addition, here we found that DDX23 is a nucleus-located dsRNA sensor. It has been appreciated for many years that the nucleus is “immune privileged” and the intracellular surveillance of viral nucleic acids occurs exclusively in cytosolic and endosomal compartments. However, evidence has challenged this dogma (38, 39). For example, the interferon-γ-inducible protein16 (IFI16) is predominantly localized in the nucleus and has been shown to be a nuclear PRR to sense nuclear viral DNA (40–43). Given that some RNA viruses such as influenza viruses replicate in the nucleus of their host cells and shuttling of viral RNAs between nucleus and cytoplasm is the key step of their life cycle (44–46), it is rational that constitutively expressed RNA sensors are also existed in the nucleus. Thus, DDX23, a resident nuclear helicase which can translocate to a cytoplasm upon poly(I:C) stimulation, may sense dsRNA both in nucleus and cytoplasm.

### DDX23 Pairs With TRIF or MAVS to Trigger Antiviral Responses

Here, we have found that human DDX23 senses the RNA virus and triggers antiviral signals by interacting with TRIF or MAVS. The N-terminal region of DDX23 was required for its binding to poly(I:C) and sensing RNA virus, while the DExD/H domain was required for DDX23-TRIF and/or DDX23-MAVS interactions. Similarly, DHX9 binds poly(I:C) through the N-terminal DSRM domain, but not the DExD/H nor HelicC domains (6). However, our endogenous IP data indicated that the DDX23-TRIF-MAVS complex does not exist in both resting and stimulated cells, suggesting that TRIF and MAVS do not share a common signaling pathway in transferring antiviral signals from DDX23. A study has also shown that RLRs activate cellular immune responses independent of MAVS signaling (47). Thus, we can assume that many immune related DExD/H helicases may utilize more than one adaptor for downstream signaling transduction, such as RIG-I and DDX23. Moreover, both DDX23-TRIF and DDX23-MAVS complexes exist in resting cells, suggesting that these complexes may represent an early sensor of poly(I:C) that triggers the initial IFN production. Thus, DDX23 may perform initial RNA sensing both in nucleus and cytosol by forming complex with TRIF or MAVS.

## Conclusion

Although several DExD/H helicases have been shown to play important roles in antiviral immunity in mammals and insects, our understanding of this family in antiviral immunity in basal chordate is only beginning to emerge and remains largely un-explored. The present study suggests that DExD/H helicases, such as DHX9, DHX15, and DDX23, play essential roles in recognizing dsRNA across species. We also identified a nuclear dsRNA sensor, DDX23, which pairs with TRIF or MAVS to trigger antiviral immune responses in mammals. These results suggest that evolutionary pressure may have led the innate immune system to develop different and redundant sensors for detecting RNA species in viral infection.

## Data Availability

The raw RNA sequencing data has been deposited to NCBI GEO database and assigned as GSE132847.

## Author Contributions

SY and JR conceived and designed the study and drafted the manuscript. JR and YC completed most of the experiments. TL performed the detection of ISGs. PL and SW performed the Co-IP and reporter assays. DP performed the RT-PCR assays. YW and XJ performed flow cytometry analyses. SY, JR, SC, and AX reviewed and edited the manuscript. SY and AX approved the submitted manuscript.

### Conflict of Interest Statement

The authors declare that the research was conducted in the absence of any commercial or financial relationships that could be construed as a potential conflict of interest.
