# Supplementary material for: DDX23, an Evolutionary Conserved dsRNA Sensor, Participates in Innate Antiviral Responses by Pairing With TRIF or MAVS
Source: Front Immunol. 2019 Sep 18;10:2202. doi: 10.3389/fimmu.2019.02202 (PMC6759578; doi:10.3389/fimmu.2019.02202)

Supplemental Material

Supplemental Figure 1. Domain topology of several members of amphioxus DExD/H helicases. Amphioxus DDX1, DDX3, DDX17, DDX24, DHX33, DDX41, SKIV2L and DICER showed high domain conservations with their human counterparts. DEXDc domain (DEAD-like helicases superfamily), HELICc domain (helicase superfamily c-terminal domain), DSRM domain (Double-stranded RNA binding motif), HA2 domain (Helicase associated domain (HA2), AAA domain (ATPases associated with a variety of cellular activities), SPRY domain (domain in SPla and the RYanodine Receptor), ZnF_C2HC domain (zinc finger), DSHCT domain (C terminal domain in DOB1/SK12/helY-like DEAD box helicases), PAZ domain (the proteins Piwi Argonaut and Zwille), RIBOc domain (Ribonuclease III family).

Supplemental Figure 2. (A) *In Vitro* pull-down assays of poly(I:C), poly(C) and ISD binding to V5-tag DmDDX23 in transfected *Drosophila* S2 cell lysates. (B) Immunoblotting of endogenous DDX23 and MAVS in A549 cells with indicated antibodies. A549 cells were transfected with siRNA (control) or with siRNA targeting DDX23 (si-DDX23), or MAVS (si-MAVS). (C-M) qRT-PCR analysis showed that knockdown human DDX23 results in the reduced transcription of *IFN-β, IFN-α, IL-6, TNF-α, RANTES , CXCL10, RIG-I, MDA5, MxA, OAS1 and IFIT2* in cells stimulated with poly(I:C) (LMW) delivered with Lipofectamine 2000 or infected with VSV for 12 h. (N-O) Effects of DDX23 silencing on transcription of *IFN-β and IFIT2* induced by HSV-1 infection. Mean ± SEM; *p < 0.05, **p < 0.01, Student's t test.

Supplemental Figure 3. (A) KEGG analysis of differentially expressed genes in uninfected scrambled siRNA (siControl) versus DDX23 knockdown (siDDX23) cells. (B) KEGG analysis of differentially expressed genes in VSV-infected scrambled siRNA (siControl) versus DDX23 knockdown (siDDX23) cells. Enriched antiviral signaling pathways upon viral infection are highlighted in red.

Supplemental Figure 4. (A) Full length, DDX23-A or MAVS was overexpressed in A549 cells and then cells were infected by VSV-eGFP. FACS analysis of the GFP-positive cells and western blotting using anti-VSV Glycoprotein were shown. Mean ± SEM; *p < 0.05, **p < 0.01, Student's t test. (B) Immunoblot analysis of TBK1, MyD88 and MAVS in A549 cells transfected with control siRNA or siDDX23 and stimulated for 8hr with poly(I:C) or VSV. (C) The expression of MxA was detected by RT-PCR in control and DDX23 silencing A549 cells after VSV infection. (D) The splicing pattern of MxA mRNAs was determined by RT-PCR in control and DDX23 silencing A549 cells.

Supplemental Figure 5. (A) Full length or DDX23-A, B, C and D was overexpressed in A549 cells. None of DDX23-FL or its truncated mutants has effort on the activation of NF-κB and IFN-response. (B-E) DDX23-A and DDX23-B could mount the activation of NF-κB and IFN responses mediated both by MAVS and TRIF, but mutants of DDX23-C and DDX23-D could not make the same efforts. (F)

Characterization of RIG KO and MAVS KO 293T cells. 293T WT or MAVS KO cells were left unstimulated, or stimulated with poly(I:C) (3μg/mL) for 10 hr. Expression levels of RIG-I, MAVS and β-actin were monitored by immunoblotting.

Supplemental Figure 1

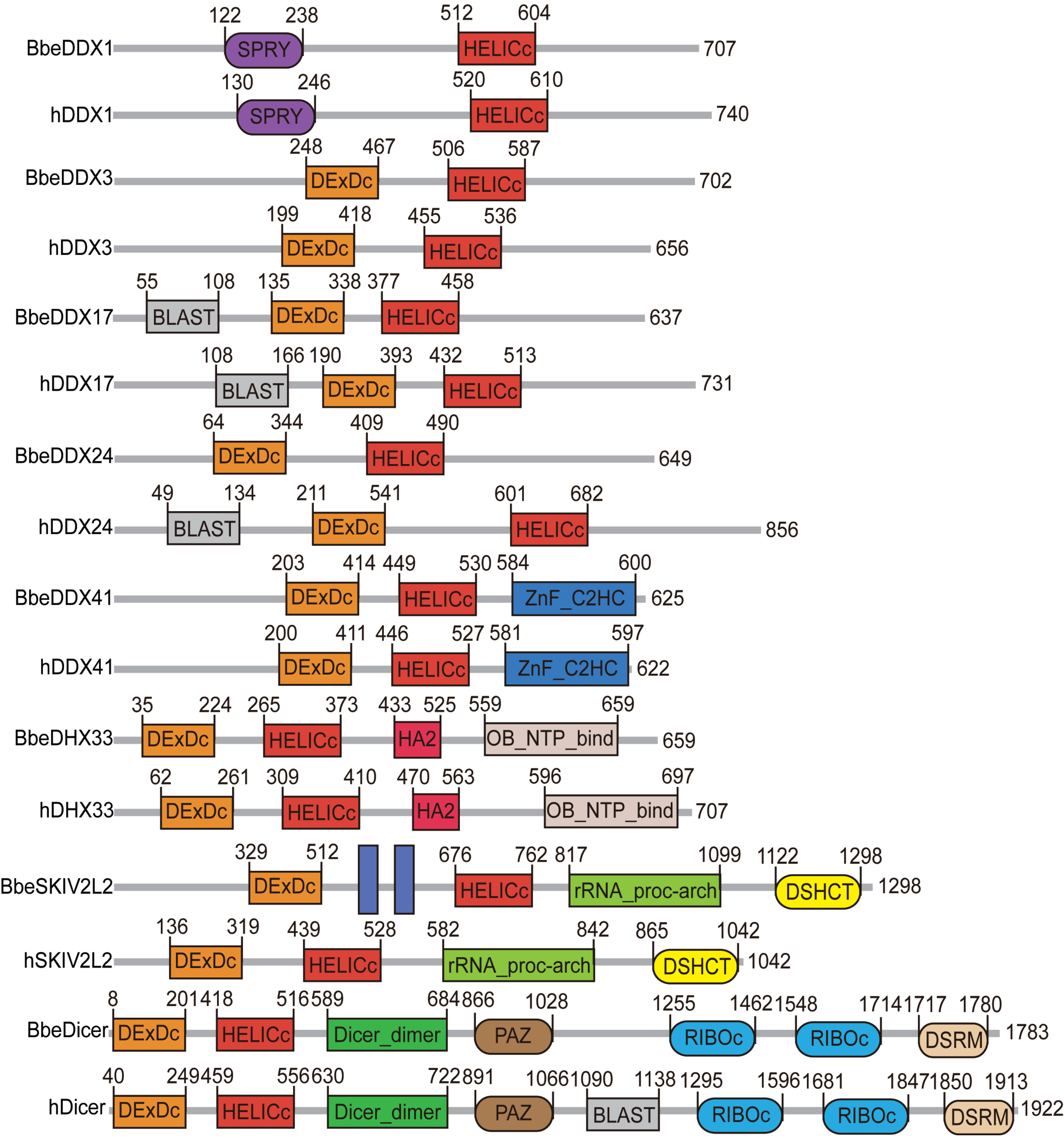

Supplemental Figure 2

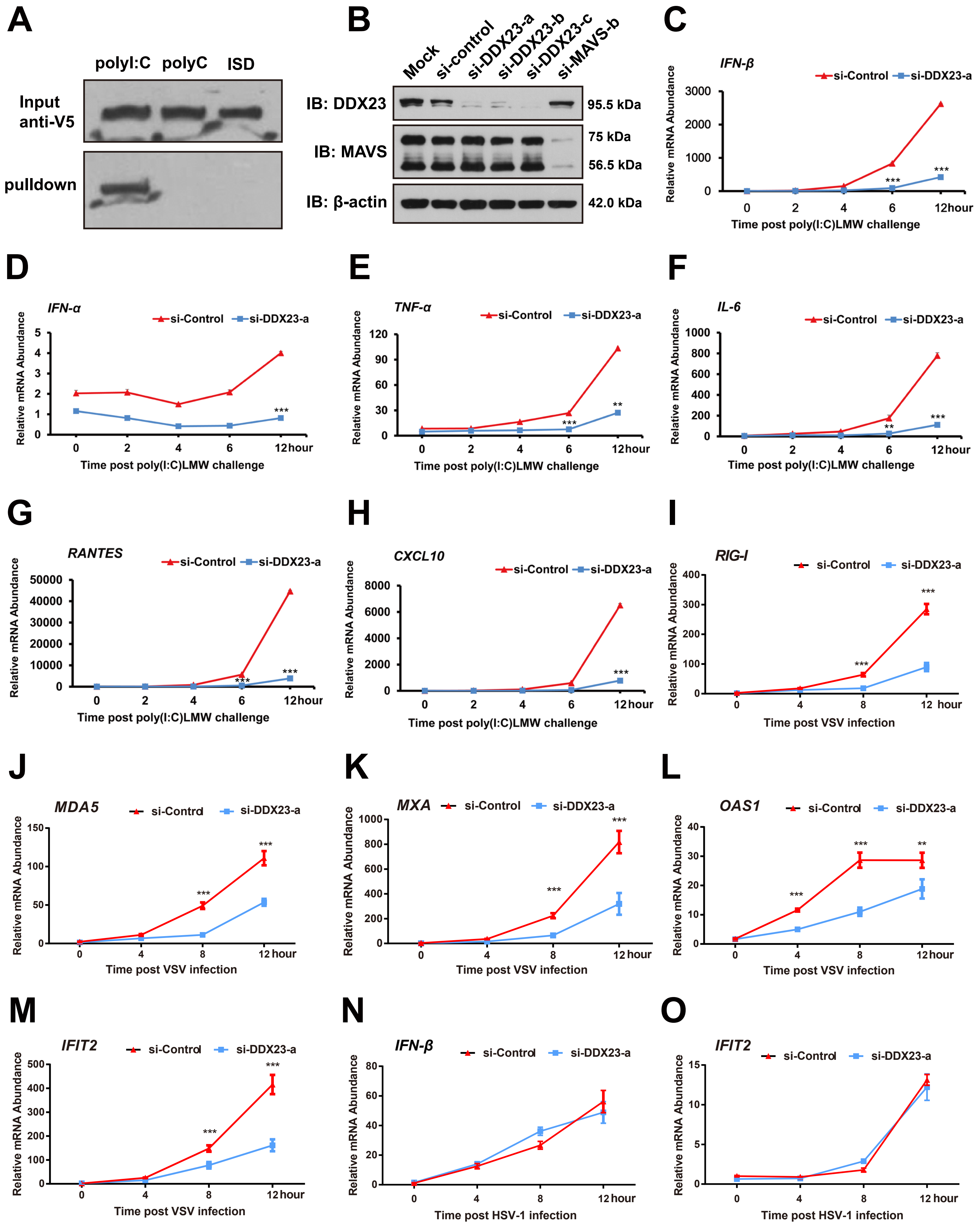

Supplemental Figure 3

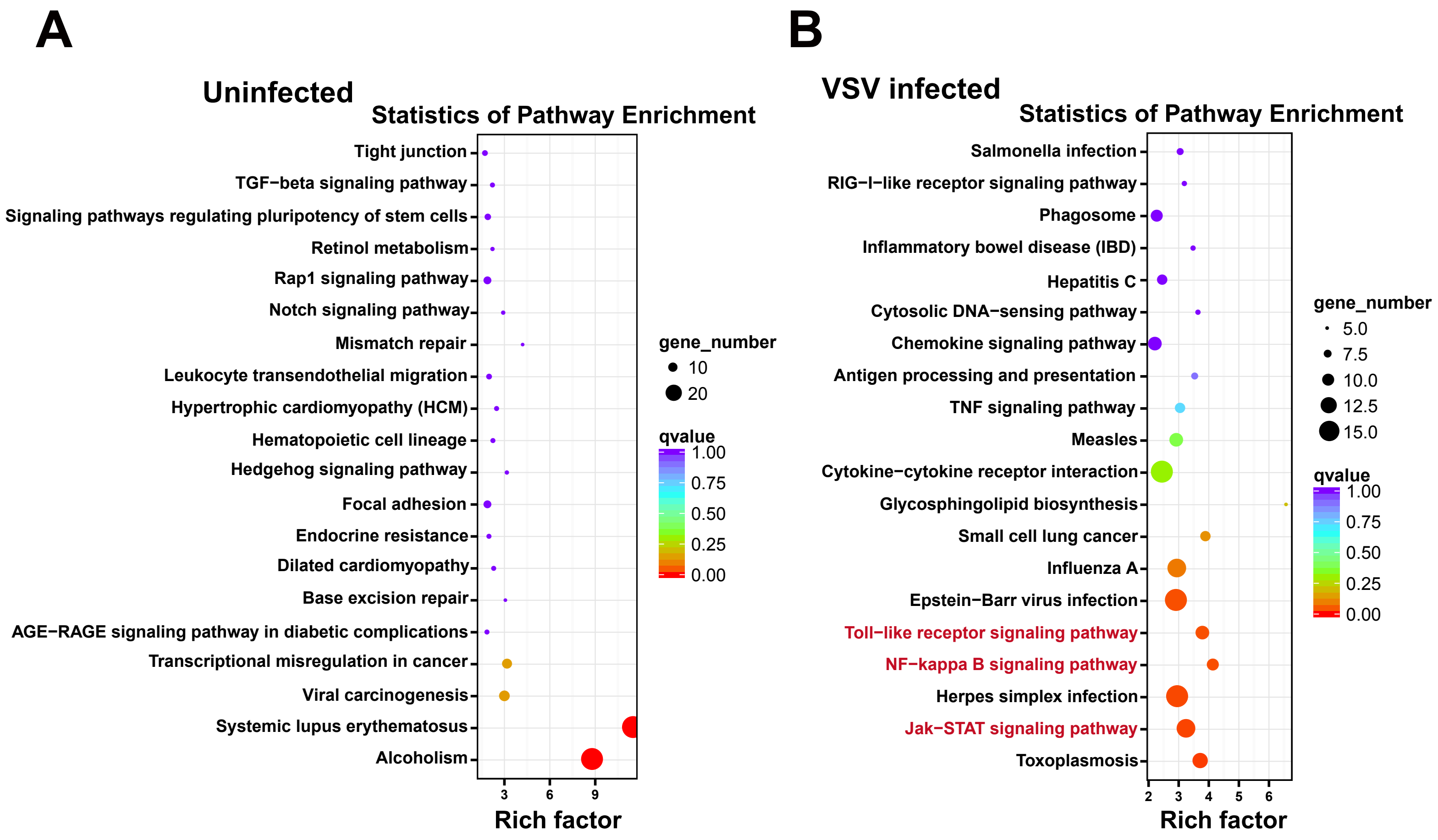

Supplemental Figure 4

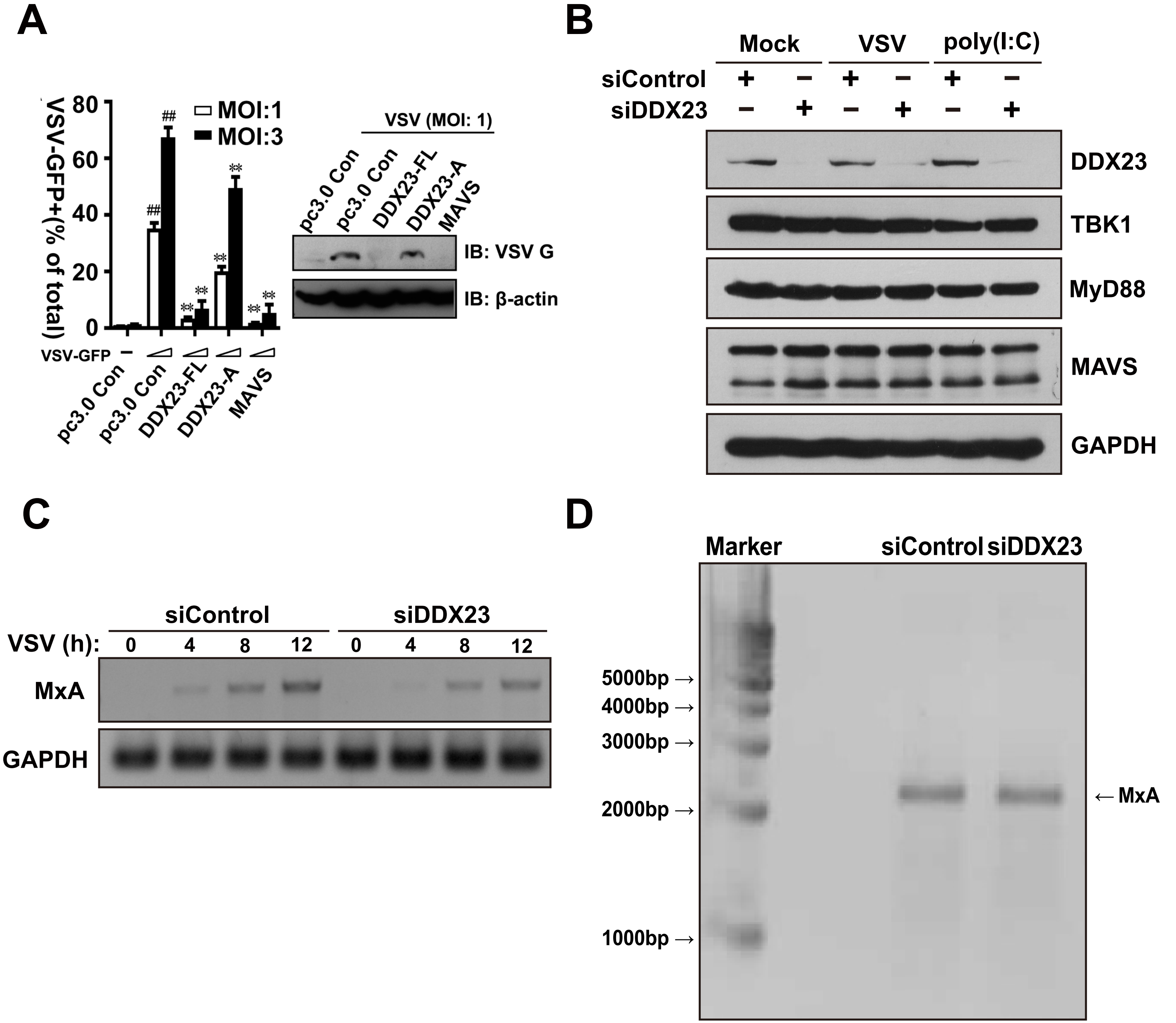

Supplemental Figure 5

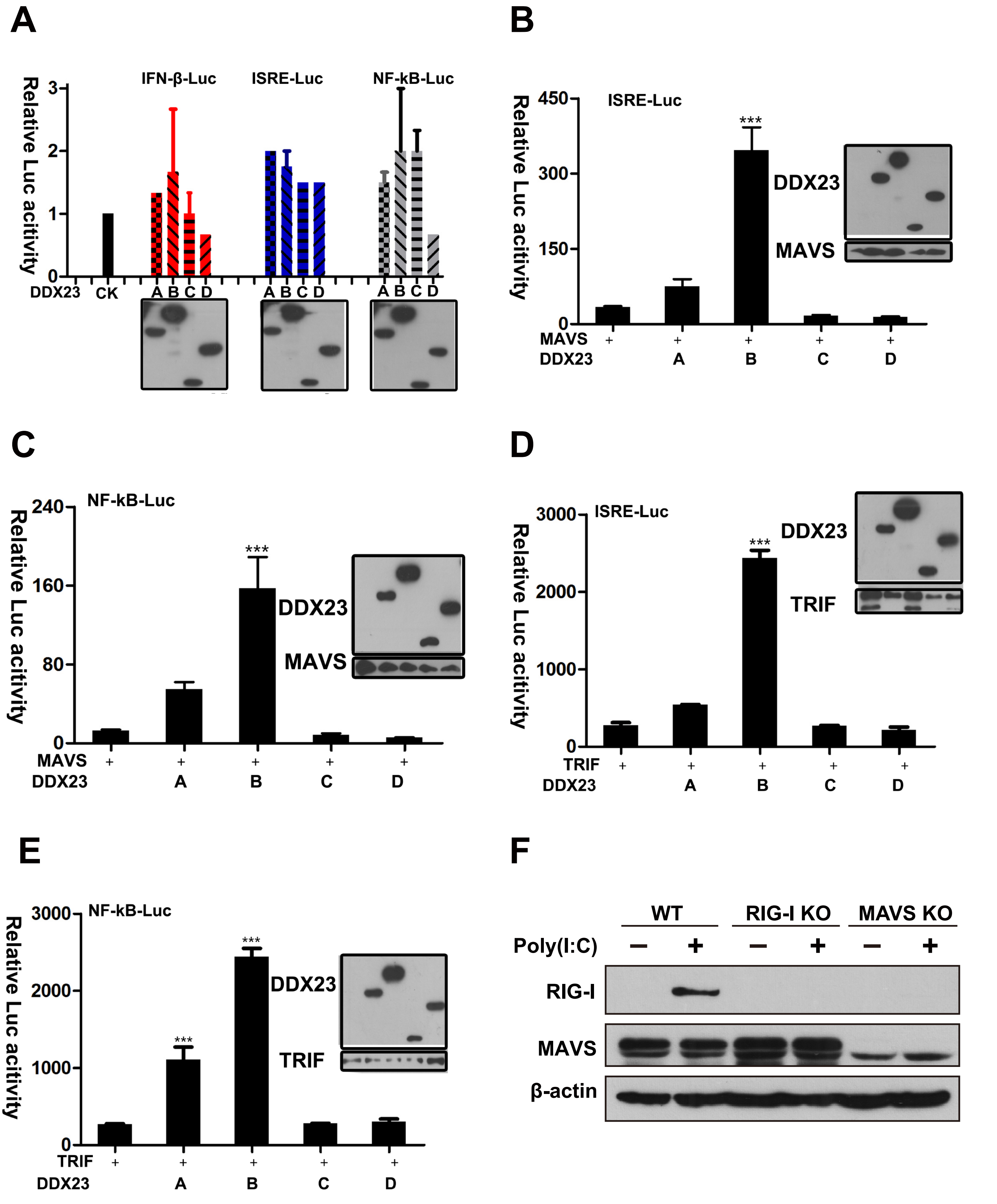

Supplement: Supplementary file 1 [file Data_Sheet_1.pdf]
